# Intraocular Pressure-Induced Endothelial Dysfunction of Retinal Blood Vessels Is Persistent, but Does Not Trigger Retinal Ganglion Cell Loss

**DOI:** 10.3390/antiox11101864

**Published:** 2022-09-21

**Authors:** Maoren Wang, Hanhan Liu, Ning Xia, Huige Li, Tim van Beers, Adrian Gericke, Verena Prokosch

**Affiliations:** 1 Department of Ophthalmology, University Medical Center, Johannes Gutenberg University Mainz, Langenbeckstr. 1, 55131 Mainz, Germany; 2 Department of Ophthalmology, Faculty of Medicine and University Hospital of Cologne, University of Cologne, 50937 Cologne, Germany; 3 Department of Pharmacology, University Medical Center, Johannes Gutenberg University Mainz, Langenbeckstr. 1, 55131 Mainz, Germany; 4 German Center for Cardiovascular Research (DZHK), Partner Site Rhine-Main, 55131 Mainz, Germany; 5 Molecular Cell Biology, Institute I for Anatomy, University of Cologne Medical School, 50931 Cologne, Germany

**Keywords:** glaucoma, retinal arterioles, vascular endothelial dysfunction, oxidative stress, retinal ganglion cells

## Abstract

Research has been conducted into vascular abnormalities in the pathogenesis of glaucoma, but conclusions remain controversial. Our aim was to test the hypothesis that retinal endothelial dysfunction induced by elevated intraocular pressure (IOP) persists after IOP normalization, further triggering retinal ganglion cell (RGC) loss. High intraocular pressure (HP) was induced in mice by episcleral vein occlusion (EVO). Retinal vascular function was measured via video microscopy in vitro. The IOP, RGC and their axons survival, levels of oxidative stress and inflammation as well as vascular pericytes coverage, were determined. EVO caused HP for two weeks, which returned to baseline afterwards. Mice with HP exhibited endothelial dysfunction in retinal arterioles, reduced density of RGC and their axons, and loss of pericytes in retinal arterioles. Notably, these values were similar to those of mice with recovered IOP (RP). Levels of oxidative stress and inflammation were increased in HP mice but went back to normal in the RP mice. Our data demonstrate that HP induces persistent endothelial dysfunction in retinal arterioles, which persists one month after RP. Oxidative stress, inflammation, and loss of pericytes appear to be involved in triggering vascular functional deficits. Our data also suggest that retinal endothelial dysfunction does not affect RGC and their axon survival.

## 1. Introduction

Glaucoma is composed of a heterogenous group of diseases characterized by progressive degradation of retinal ganglion cells (RGC) and their axons, causing irreversible loss of vision and eventually blindness [1,2]. The pathogenesis of glaucoma is still not well known. There are two major theories for explaining the disease: the mechanical and vascular theories. The mechanical theory posits that elevated IOP causes distortion of the lamina cribrosa plates and interruption of the axoplasmic flow, leading to the degradation of RGCs [3]. However, this does not explain why ocular hypertension patients lack glaucoma [4] and normal tension glaucoma (NTG) patients present glaucomatous abnormalities although their intraocular pressure (IOP) values are normal [5]. Moreover, the mechanical theory cannot provide a sufficient explanation for the fact that patients show deterioration in visual fields despite low IOP [6]. The vascular theory tries to explain glaucoma as a consequence of insufficient blood supply, but this theory remains controversial [5,7]. In one of our previous studies, we demonstrated that moderately elevated IOP causes endothelial dysfunction and impaired autoregulation in retinal arterioles [8]. Reactive oxygen species and inflammation within the retinal tissue, caused by elevated IOP, seem to contribute to dysfunctional vessels [8]. However, to the best of our knowledge, no study has yet demonstrated whether retinal vascular dysfunction recovers after the normalization of IOP and, if not, whether a persistent endothelial dysfunction and impaired autoregulation can lead to further neurodegeneration even after IOP normalization, explaining further RGC loss in patients despite IOP normalization. In the present study, we hypothesized that retinal endothelial dysfunction persists after IOP normalization, further triggering glaucomatous neurodegeneration. This hypothesis would explain the vascular involvement in further RGC loss despite IOP normalization.

## 2. Materials and Methods

### 2.1. Animals

All animal experiments were conducted in accordance with the EU Directive 2010/63/EU for animal experiments and the Association for Research in Vision and Ophthalmology (ARVO) guidelines. The use of animals in this study was approved by the ethics committee of animal research Rhineland-Palatinate, Germany (permission number: 14-1-085). C57 BL/6J male mice at the age of 8–9 weeks were used in the experiments. The standard condition of housing mice was used as follow: 12 h light/dark cycle, temperature of 22 ± 2 °C, humidity of 55 ± 10%, and free access to food and water.

### 2.2. Experimental Design

All animals received sham surgeries or episcleral vein occlusion (EVO) treatments in their right eyes. After this, they were divided into three groups: the control group, in which mice received sham surgery only, the EVO (HP) group, in which mice underwent EVO and were sacrificed two weeks after IOP elevation, and the EVO (RP) group, in which mice were sacrificed four weeks after IOP normalization. After the surgeries, we compared the following changes among the three groups: IOP values, retinal vascular function, RGC density, optic nerve axon density, retinal oxidative stress, and inflammatory levels, as well as pericytes coverage on retinal blood vessels.

### 2.3. IOP Monitoring

The IOP of the mice was monitored using a TonoLab rebound tonometer (iCare, Vantaa, Finland) in animals that were awake, as previously described [9,10]. All measurements were conducted between 9:00 a.m. to 12:00 p.m. for comparability. After six consecutive measurements, the tonometer generated a mean IOP. We took 5 sets of measurements and averaged these IOP values.

### 2.4. Induction of Glaucoma

The glaucoma mouse model was induced by occlusion of the three episcleral veins in their right eyes, as previously described [8,11]. Briefly, mice were anesthesized using a ketamine (100 mg/kg) and xylazine (10 mg/kg) solution via intraperitoneal injection. We also applied one drop of the 4 mg/mL oxybuprocainhydrochloride (Novesine^®^ 0.4% Eyedrops, OmniVision^®^, OmniVision GmbH, Puchheim, Germany) onto the scathe ocular surface for local anesthesia. Subsequently, an incision was made on the conjunctiva and Tenon’s capsule to expose the episcleral veins. The major trunks of the veins were cauterized using an ophthalmic thermal cautery (Fine Science Tools GmbH, Heidelberg, Germany); following this, the remaining tissue of the trunk was cut. Finally, the conjunctiva was put back to its original location and ofloxacin ointment was given onto the ocular surface to prevent inflammation. Sham surgery was conducted in a similar way but without causing damage to the episcleral veins.

### 2.5. Measurement of Vascular Reactivities in Retinal Arterioles

The retinal vascular reactivities were measured in vitro as described in previous studies [12,13]. Briefly, mice (*n* = 8 per group) were sacrificed by carbon dioxide overdose. Retinas with ophthalmic arteries from right eyes were carefully isolated. The retinal vessels were pressurized via ophthalmic arteries that were perfused using a Krebs buffer via a micropipette connected to a silicone tube. The intraluminal pressure of the retinal vessels could be adjusted by changing the fluid level in the tube. The retinas were mounted on a transparent platform and fixed to its bottom by a steel ring. This platform was immersed in a reservoir filled with Krebs buffer. The mixture of gas (95% O_2_ and 5% CO_2_) was bumped into the reservoir and the temperature of the fluid was controlled at 37 °C. After being equilibrated for 30 min, the function of the first-order retinal arterioles was measured. The diameter changes in the retinal arterioles in response to different intraluminal pressure levels and various vasoactive agents were visualized using a microscope and recorded by a video camera. Autoregulation of the retinal arterioles was measured when intraluminal pressure was increased gradually from 10 mmHg to 80 mmHg, while retinal vascular function was investigated in response to the thromboxane mimetic, U46619 (10^−11^ M to 10^−6^ M; Cayman Chemical, Ann Arbor, MI, USA), the endothelium-dependent vasodilator, acetylcholine (10^−9^ M to 10^−4^ M; Sigma-Aldrich, Munich; Germany), and the endothelium-independent NO donor, sodium nitroprusside (10^−9^ M to 10^−4^ M; Sigma-Aldrich), at an intraluminal pressure of 50 mmHg. The vessels were pre-constricted to ≈50% of their initial diameter by U46619 before measuring the reactivities to acetylcholine and nitroprusside.

### 2.6. Quantification of Reactive Oxygen Species (ROS)

ROS levels were determined in DHE (1 μM)-stained retinal cryosections of 10 μm thickness, as previously reported [8,14]. The retinal cryosections were imaged using the Zeiss Imager M.2 equipped with an Apotome.2 (Carl Zeiss; Jena, Germany).

### 2.7. Immunostaining on Retinal Cryosections

NOX2 immunoreactivity was determined on retinal cryosections (10 μm), as previously reported [8]. Briefly, tissue sections were fixed in 4% paraformaldehyde for 20 min, followed by being washed with PBS 5 min for three times. Then, retinas were blocked with 1% bovine serum albumin (BSA) for 30 min before being incubated with a primary antibody directed against NOX2 (1:200, ab129068, Abcam, Cambridge, UK) for 2 h at room temperature. After being washed by PBS 5 min for 3 times, all the steps were conducted in the dark. The secondary antibody (goat anti-rabbit IgG H + L; Alexa Flour 546; 1:1000, A11010, Life Technologies, Carlsbad, CA, USA) was used. After being washed with PBS 5 min for 3 times, slides were mounted using VECTASHIELD ^®^ Mounting Medium with DAPI (BIOZOL Diagnostica Vertrieb GmbH, Eching, Germany). Photos were taken using the Zeiss Imager M.2 equipped with Apotome.2 (Carl Zeiss; Jena, Germany).

### 2.8. Quantification of Gene Expression by Real-Time PCR

Messenger RNA expression of *NOX2* and *TNF-α* was determined by real-time PCR [15]. Briefly, total RNA was isolated from the retina samples using peqGOLD TriFast™ (PEQLAB), and cDNA was generated with the High Capacity cDNA Reverse Transcription Kit (Applied Biosystems, Darmstadt, Germany). Real-time PCR reactions were performed on a StepOnePlus™ Real-Time PCR System (Applied Biosystems) using SYBR^®^ Green JumpStart™ Taq ReadyMix™ (Sigma-Aldrich) and 20 ng cDNA. The relative mRNA levels of the target genes were quantified using a comparative threshold (C_T_) normalized to the TATA-binding protein (TBP) housekeeping gene. Messenger RNA expression is presented as the fold-change relative to control. The PCR primer sequences were *TNF-α*_Forward: GCC TCT TCT CAT TCC TGC TTG, *TNF-α*_ Reverse: CTG ATG AGA GGG AGG CCA TT; *NOX2*_Forward: CCA ACT GGG ATA ACG AGT TCA, *NOX2*_Reverse: GAG AGT TTC AGC CAA GGC TTC; *TBP*_Forward: CTT CGT GCA AGA AAT GCT GAA T, *TBP*_Reverse: CAG TTG TCC GTG GCT CTC TTA TT [15].

### 2.9. Immunostaining on Retinal Flatmounts

Immediately after sacrificing the mice, the right eyes of mice were enucleated and transferred into cooled phosphate-buffered saline (PBS) (Gibco, Thermo Fisher, Waltham, MA, USA). Then, the retinas were isolated and immersed into 4% paraformaldehyde (Histofix, Roth, Karlsruhe, Germany) for 30 min. Subsequently, the retinas were washed with PBS for 10 min twice, followed by dehydration in a 30% sucrose solution for 24 h at 4 °C. Next, the retinas were frozen in liquid nitrogen and stored at −80 °C for further analysis. After being thawed and washed with PBS for 10 min twice, the retinas were incubated in a blocking solution containing 10% fetal calf serum (FCS) and 0.3% Triton-X-100 in PBS for 2 h at room temperature. Then, either the primary antibody Brn3a (1:200, MAB1585, EMD Millipore Corporation, Temecula, CA, USA) was used as an RGC marker [16], or α-SMA (1:400, ab124964, abcam, Cambridge, UK) was used as a pericytes marker [17,18] to incubate the retinas overnight at 4 °C. Next, the retinas were washed with PBS and incubated with the secondary antibody (goat anti-mouse IgG H + L; Alexa Flour 488; 1:1000 in 10% FCS-PBS, ab150113, abcam, Cambridge, UK) or the secondary antibody (goat anti-rabbit IgG H + L; Alexa Flour 546; 1:1000 in 10% FCS-PBS, A11010, Life Technologies, Carlsbad, CA, USA) for 2 h at room temperature. Later, the retinas were washed twice with PBS and mounted on slides. Finally, a drop of mounting medium was added onto the retina, and a coverslip was put over it. Photos were taken by Zeiss Imager M.2 equipped with Apotome.2 (Carl Zeiss; Jena, Germany). RGC numbers and fluorescence intensity of pericytes were quantified using ImageJ software (http://rsb.info.nih.gov/ij/ (accessed on 18 April 2022), NIH, Bethesda, MD, USA).

### 2.10. Assessment of Optic Nerve Degeneration

Optic nerve degeneration was assessed by p-phenylenediamine (PPD)-stained optic nerve cross sections, as previously described [19,20]. The semi-thin cross sections of optic nerves were taken from 1.0 mm posterior to the eyeballs. PPD stains the myelin sheaths of the optic nerve and the darker axoplasm of sick or dying axons. The sections were imaged under a Zeiss Axio Imager Z1 Microscope (Zeiss, Oberkochen, Germany) with a Zeiss Plan-ACHROMAT 100× Lens (Zeiss, Oberkochen, Germany). Image Acquisition was performed using a Canon EOS 6D Mk II camera (Canon, Krefeld, Germany) and Canon EOS Utility Software (Canon, Krefeld, Germany). The degeneration of the optic nerves was evaluated using the optic nerve damage score, as previously reported [21,22].

### 2.11. Statistic Analysis

Data are expressed as the mean ± SEM, and *n* represents the number of animals in each group. Comparison of IOP, autoregulation, and vascular responses to different vasoactive agents were made using two-way ANOVA. RGC density, the optic nerve damage score, ROS levels, mRNA expression levels, and immunostaining intensity were compared using one-way ANOVA. For comparison of multiple groups, either the Sidak’s multiple comparisons test or the Tukey’s multiple comparisons test was used.

## 3. Results

### 3.1. EVO Induced Reversible IOP Elevation

**Figure 1:**
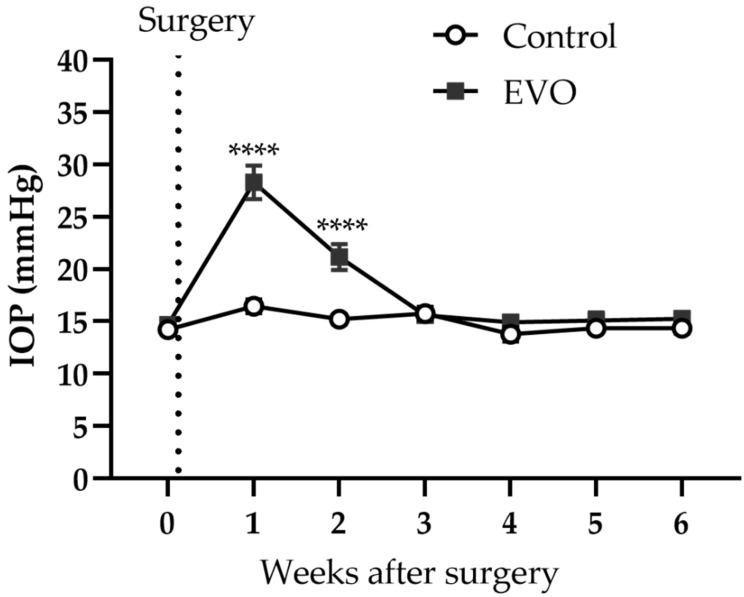
Follow up of IOP throughout the whole experiment. EVO increased IOP for two weeks. Control group refers to the sham group. Values are expressed as mean ± SEM (**** *p* < 0.0001; *n* = 8 per group).

Before EVO and sham surgery, the IOP baseline values were similar between the two groups of mice. EVO caused IOP elevation in mice for two weeks. However, three weeks after EVO, IOP had already normalized (Figure 1). One week after surgery, the mean IOP of the mice in the EVO group was 28.3 ± 1.5 mmHg and, as such, significantly higher than that (16.4 ± 0.6 mmHg) of the sham group (**** p < 0.0001). Two weeks after surgery, the mean IOP of mice in the EVO group was still significantly higher than that of the sham group (21.2 ± 1.1 versus 15.2 ± 0.4, EVO versus Sham; **** p < 0.0001). The IOP values remained unchanged in the mice who received sham surgery throughout the whole experiment.

### 3.2. Elevated IOP Induced Persistently Abnormal Autoregulation and Endothelial Dysfunction in Retinal Arterioles

**Figure 2:**
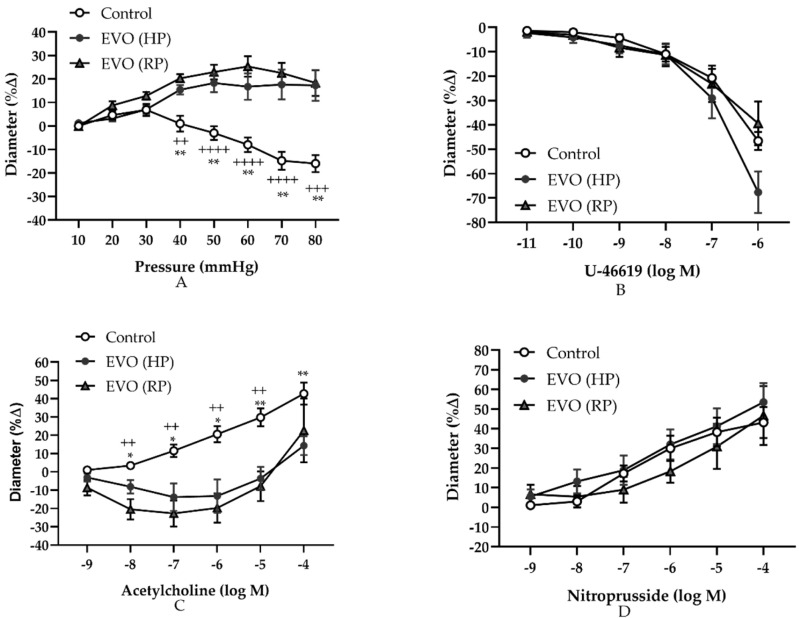
Relative changes in the luminal dimeters of retinal arterioles in response to different intravascular pressures and vasoactive agents. The changes in response to stepwise increases in intraluminal pressure (**A**), to the thromboxane mimetic, U46619 (**B**), to the endothelium-dependent vasodilator, acetylcholine, (**C**) and to the endothelium-independent vasodilator, nitroprusside (**D**). EVO: episcleral vein occlusion; HP: high intraocular pressure; RP: recovered intraocular pressure. Control group refers to the sham group. Values are expressed as mean ± SEM (* *p* < 0.05, ** *p* < 0.01, Control vs. EVO (HP); ^++^ *p* < 0.01, ^+++^ *p* < 0.001, ^++++^ *p* < 0.0001, Control vs. EVO (RP); *n* = 8 per group).

Retinal vasoreactivity responses to stepwise increases in intraluminal pressure and to different pharmacological agents are shown in the Figure 2. Retinal arterioles in the sham control group responded with a gradual constriction from 30 mmHg to 80 mmHg, which is indicative of intact autoregulation (Figure 2A). However, the vessels in both the EVO (HP) group and the EVO (RP) group responded with dilation, indicating that autoregulation was impaired (Figure 2A). The autoregulation of retinal arterioles in the HP group was similar to that of the RP group (Figure 2A). Vascular reactivities to the thromboxane mimetic 9,11-Dideoxy-9α,11α-methanoepoxy prostaglandin F2α (U46619, 10^−11^ M to 10^−6^ M) were similar between the three groups (Figure 2B). In contrast, the vasoreactivity towards the endothelium-dependent vasodilator acetylcholine was significantly attenuated in mice from the HP group and the RP group (10^−8^ M–10^−4^ M) (Figure 2C). Retinal arteriole vasodilation was similar between the three groups in response to the endothelium-independent vasodilator, sodium nitroprusside (10^−9^ M–10^−4^ M) (Figure 2D).

### 3.3. Elevated IOP Reversibly Increased the Retinal ROS Concentration

**Figure 3:**
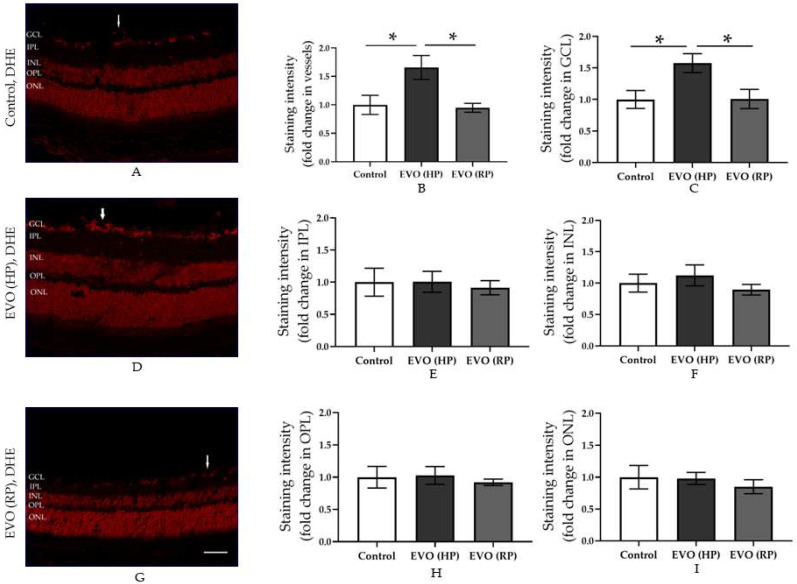
DHE-stained retinal cryosections and intensity quantification for individual retinal layers. (**A**,**D**,**G**) show the DHE-stained retinal cryosections in the control, EVO (HP), and EVO (RP) groups, respectively. (**B**,**C**,**E**,**F**,**H**,**I**) show the staining intensity quantified from vessels, GCL, IPL, INL, OPL, and ONL, respectively. The white arrows point to retinal blood vessels. GCL: ganglion cell layer; IPL: inner plexiform layer; INL: inner nuclear layer; OPL: outer plexiform layer; ONL: outer nuclear layer. EVO: episcleral vein occlusion; HP: high intraocular pressure; RP: recovered intraocular pressure. Control group refers to sham group. Values are expressed as mean ± SEM (* *p* < 0.05; *n* = 5–6 per group, scale bar = 50 μm).

The fluorescence intensity in the retinal cryosections stained with DHE showed ROS concentration in retinal tissue (Figure 3). High IOP increased ROS concentration two weeks after IOP elevation, shown as increased intensity in the ganglion cell layer including blood vessels (Figure 3B–D). However, four weeks after the normalization of IOP in the EVO (RP) group, increased ROS concentrations dropped to control levels (Figure 3B,C,G). This indicates that increased ROS concentration is IOP-dependent.

### 3.4. Elevated IOP Reversibly Increased the Expression of NOX2 in the Retina

**Figure 4:**
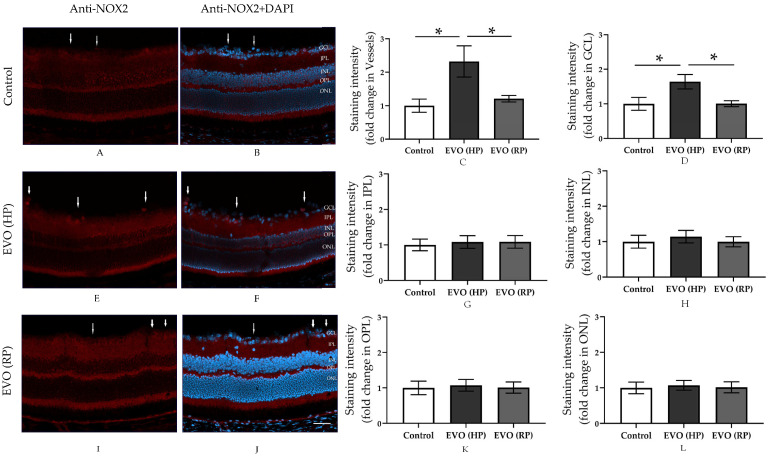
Immunostaing of anti-NOX2 and quantitative analysis of its fluorescence intensity in retinal cryosections. The anti-NOX2 stained retinal cryosections from Control group (**A**,**B**), EVO (HP) group (**E**,**F**) and EVO (RP) group (**I**,**J**). (**C**,**D**,**G**,**H**,**K**,**L**) show the staining intensity quantified from vessels, GCL, IPL, INL, OPL and ONL respectively. The white arrows point to retinal blood vessels. GCL: ganglion cell layer; IPL: inner plexiform layer; INL: inner nuclear layer; OPL: outer plexiform layer; ONL: outer nuclear layer. EVO: episcleral vein occlusion; HP: high intraocular pressure; RP: recovered intraocular pressure. Control group refers to sham group. Values are expressed as mean ± SEM (* *p* < 0.05; *n* = 6 per group, scale bar = 50 μm).

The immunoreactivity of NOX2 was increased in retinal vessels and the ganglion cell layer in the EVO (HP) group (Figure 4C–F). However, this overexpression of NOX2 normalized four weeks after IOP normalization, as is shown in the staining of the EVO (RP) group (Figure 4C,D,I,J).

### 3.5. Elevated IOP Reversibly Increased the Expression of Messenger RNA of NOX2 and TNF-α

**Figure 5:**
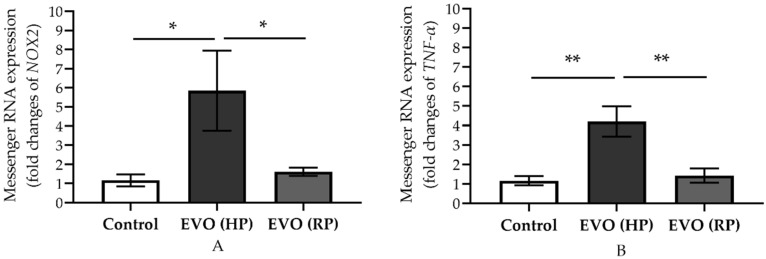
Messenger RNA expression of prooxidant gene (*NOX2*; **A**) and proinflammatory cytokine (*TNF-α*, **B**) in retina. EVO: episcleral vein occlusion; HP: high intraocular pressure; RP: recovered intraocular pressure. Control group refers to the sham group. Values are expressed as mean ± SEM (* *p* < 0.05, ** *p* < 0.01; *n* = 5–6 per group).

High IOP increased mRNA expression for the prooxidant gene, *NOX2*, and for the proinflammatory cytokine, *TNF-α*, two weeks after IOP elevation, as shown in the EVO (HP) group; the levels increased 5.9-fold (* p < 0.05) and 4.2-fold (** p < 0.01), respectively, compared to controls (Figure 5). However, these increased values dropped to control levels four weeks after IOP normalization in the EVO (RP) group (Figure 5).

### 3.6. Elevated IOP Reduced the Pericytes Coverage in the Retinal Arterioles

**Figure 6:**
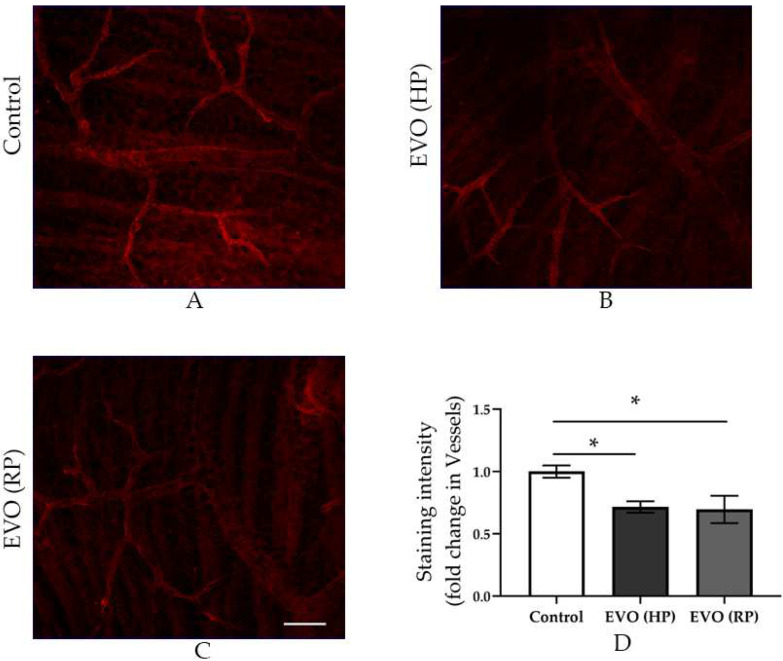
Immunostaining of anti-α-SMA and quantitative analysis of its fluorescence intensity in retinal flatmounts. The immunostained retinal flatmounts from the control group (**A**), the EVO (HP) group (**B**), and the EVO (RP) group (**C**). The staining intensity comparison among three groups (**D**). EVO: episcleral vein occlusion; HP: high intraocular pressure; RP: recovered intraocular pressure. Control group refers to the sham group. Values are expressed as mean ± SEM (* *p* < 0.05; *n* = 6 per group, scale bar = 50 μm).

The pericytes in retinal flatmounts were immunostained using an antibody directed against α-SMA (Figure 6). Increased IOP reduced the pericyte coverage in glaucoma mice with HP (Figure 6B,D). The reduced pericytes in the retinal arterioles neither decreased nor increased four weeks after IOP normalization (Figure 6C,D).

### 3.7. Elevated IOP Impaired RGC Survival

**Figure 7:**
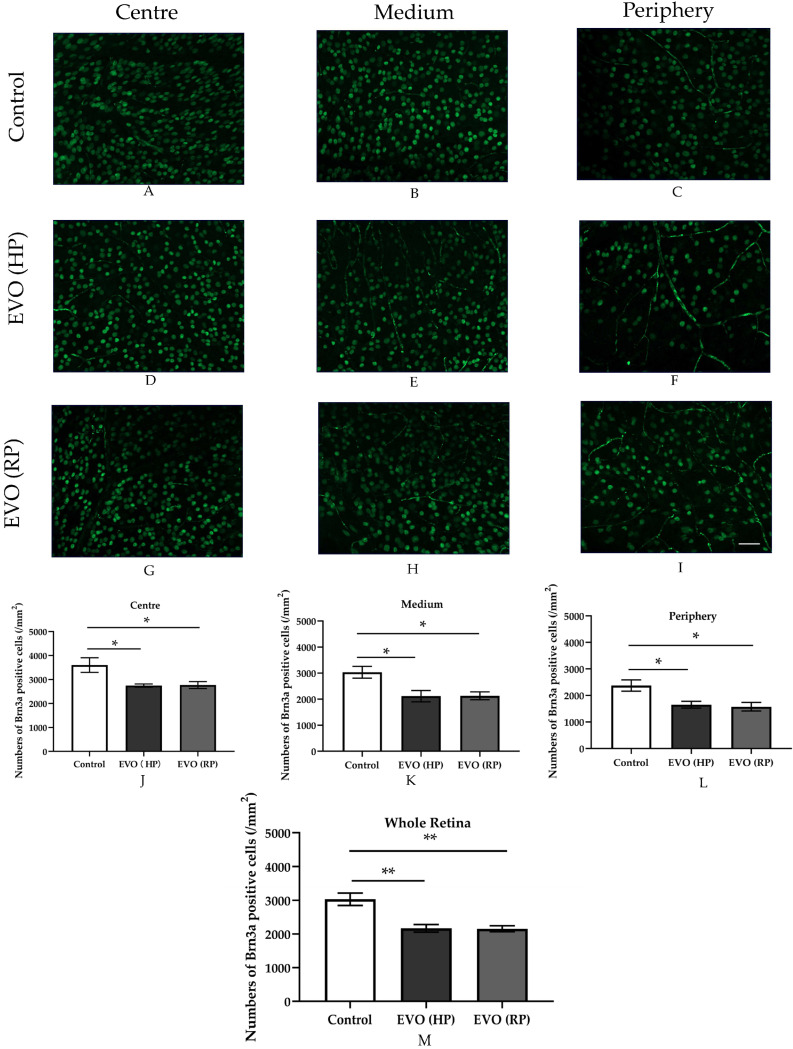
Immunostaining and RGC density in the retinal flatmounts. (**A**,**D**,**G**,**J**) show the RGC density in the center of the retina. (**B**,**E**,**H**,**K**) exhibit the RGC density in the middle of the retina. (**C**,**F**,**I**,**L**) show the RGC density in the periphery of the retina. (**M**) represents the mean RGC density in the whole retina, averaged from the three different parts. Control group refers to the sham group. Values are expressed as mean ± SEM. (* *p* < 0.05, ** *p* < 0.01; *n* = 5–6 per group, scale bar = 50 μm).

RGC survival (Figure 7) was presented as Brn3a-positive RGC density (/mm^2^) in retinal flatmounts. Compared to the sham groups, a significantly reduced RGC density was found in glaucoma mice from the EVO (HP) group, in which mice were sacrificed two weeks after EVO treatment (HP), and from the EVO (RP) group with normalized IOP, in which mice were sacrificed six weeks after EVO treatment. Notably, RGC density did not reduce further in the RP group compared to the HP group, indicating that there was no progression of RGC loss after the normalization of IOP.

### 3.8. Elevated IOP Caused Optic Nerve Damage

**Figure 8:**
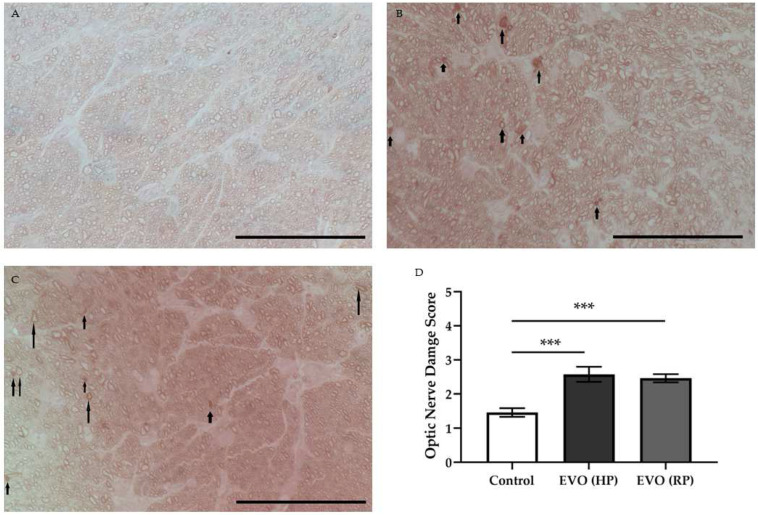
Scoring of PPD-stained optic nerve cross-sections. The normal stained axons in the control mice (**A**). The collapsed and more darkly stained abnormal axons in glaucoma mice from both the EVO (HP) group (**B**) and the EVO (RP) group (**C**). Arrows point to the abnormal axons. Higher scores were assessed both in glaucoma mice with HP and those with RP (**D**). EVO: episcleral vein occlusion; HP: high intraocular pressure; RP: recovered intraocular pressure. Control group refers to the sham group. Values are expressed as mean ± SEM. (*** *p* < 0.001; *n* = 5–6 per group, scale bar = 50 μm).

The degeneration of the optic nerve was evaluated using the optic nerve damage score, as reported previously [21,22]. EVO caused a higher optic nerve damage score in glaucoma mice both with HP (2.6 ± 0.2) and with RP (2.5 ± 0.1) compared to the control mice (1.5 ± 0.1) (*** p < 0.001) (Figure 8). Again, there was no significant difference between glaucoma mice with HP and those with RP in terms of optic nerve damage. This indicates that the optic nerve damage caused by elevated IOP did not worsen after normalization of IOP.

## 4. Discussion

Several findings emerged from this study. First, high IOP caused persistent endothelial dysfunction and impaired autoregulation in the retinal arterioles. This feature was not reversible after IOP normalization. Second, high IOP caused significant RGC and optic nerve axonal loss. Third, high IOP increased oxidative stress and NOX2 levels. Those levels were reversed after IOP normalization. Finally, increased IOP sustainably reduced the pericyte coverage in the retinal arterioles. However, neurodegeneration did not deteriorate further after the normalization of IOP, despite reduced pericyte coverage and persistent deficits in endothelial function and autoregulation.

EVO has been widely used as a method in studies of glaucoma rodents [11,23], leading to significant RGC loss to imitate neurodegeneration in glaucoma [20,24]. In a previous study of our own [8], we found that IOP elevation also impairs vascular endothelial function and autoregulation. Furthermore, in the present study, we found that vascular dysfunction due to elevated IOP seems to be sustained, because abnormal endothelial function and autoregulation did not improve in animals with recovered IOP. However, although vascular dysfunction persisted after IOP normalization, no further RGC loss was observed. This finding does not support our initial hypothesis that prolonged vascular dysfunction, apart from elevated IOP, is an additional driving force for glaucomatous damage. Apparently, endothelial dysfunction and abnormal autoregulation do not induce RGC death per se. In our previous study of mice subjected to chronic social defeat stress, retinal vascular dysfunction was also not associated with glaucomatous damage [19]. Similarly, retinal vascular dysfunction, which was observed in *ApoE^-/-^* mice, did not affect the viability of RGCs and their axons [14]. However, some studies have reached different conclusions. For example, clinical studies found that patients’ visual field defects are correlated with optic nerve head filling defects [25,26]. Similarly, glaucoma patients displayed reduced optic nerve head blood supply in optical coherence-tomography angiography [27]. These controversial conclusions may result from the different species and measuring methods, or the different durations of elevated IOP and time of glaucoma duration. Based on these findings, we cannot exclude the possibility that chronic vascular dysfunction will cause RGC damage in the long term.

Glaucoma has been reported to cause various retinal vascular abnormalities, such as decreased vessel density [28,29,30,31], reduced retinal vascular calibers [32,33,34], and modified vascular biomarkers [17,18,35,36]. Our previous study demonstrated that elevated IOP induced vascular dysfunction in retinal arterioles [8]. Increased oxidative stress levels were considered as the potential mechanism [8]. This is in line with our presented study, which shows that retinal vasodilation in response to the endothelium-dependent vasodilator acetylcholine was attenuated in glaucoma mice both with HP and RP, which is indicative of endothelial dysfunction [12].

That elevated IOP induces oxidative stress in the retina, especially in the inner retina, such as the RGC layer, has been reported in various previous animal studies [37,38,39]. This is in line with our finding that oxidative stress levels increased in HP mice. The fluorescence intensity of the DHE-stained retinal cryosection and the retinal NOX2 immunoreactivities were increased in HP animals. Oxidative stress has been reported to promote RGC death and is a risk factor for endothelial dysfunction in the aorta, and the retinal and mesenteric vessels [8,14,40,41,42,43,44,45]. Moreover, a similar trend was found regarding the proinflammatory cytokines of the *TNF-α* mRNA level. TNF-α can transmit the oxidative stress from neuron cells to vascular cells [8]. Remarkably, however, those levels decreased back to normal after the IOP normalization in the RP group. These findings suggest that oxidative stress and inflammatory responses may initiate endothelial dysfunction in retinal blood vessels, but, due to their transient increase, do not contribute to its maintenance. We thus further investigated the changes in the vascular structure.

We found the pericytes coverage was reduced in both glaucoma mice with HP and those with RP. This may explain why endothelial dysfunction of retinal arterioles still exists in glaucoma mice with RP. A growing number of studies show that there is a cross talk between endothelial cells and pericytes, so the loss of pericytes might disturb vascular endothelial function [46]. The peroxisome proliferator-activated receptor γ coactivator 1α (PGC-1α) is a known regulator of mitochondrial oxidative metabolism, and the *PGC-1α^-/-^* mice exhibit increased ROS levels [46]. The loss of pericytes may be a result of the increased oxidative stress due to elevated IOP, because the loss of pericytes was also observed in the *PGC-1α^-/-^* mice [46,47].

Autoregulation is considered to be an adaptive mechanism of vessels facing changes in perfusion pressure, which works to maintain a stable blood flow [8]. Apart from the impaired endothelial dysfunction in RP and HP mice, we observed sustainably impaired autoregulation. While stepwise increases in intraluminal pressure evoked vasoconstriction responses in retinal arterioles from sham mice at intraluminal pressures of 40–80 mmHg, retinal vasoconstriction was negligible at this pressure range in both glaucoma mice with HP and those with RP. We speculate that the impaired autoregulation of retinal arterioles is an adaptive and reactive change to the impaired endothelial function of retinal arterioles caused by increased IOP. Due to this reaction, blood flow can be increased when endothelium-dependent vasodilation is not sufficient [48].

However, there are some limitations in this study. First, we only tested RP mice four weeks after IOP normalization, so we cannot exclude the possibility that vascular function will normalize on a long-term basis. Second, there are different kinds of glaucoma mouse models with different duration of elevated IOP. We only used the EVO mice model in our present study. Therefore, the extent of impaired retinal vascular dysfunction may differ in other models. Third, we only compared the mRNA expression of *TNF-α* in proinflammatory cytokines, although one of our previous studies showed that the mRNA expression of *IFN-γ, IL-1β, IL-2, and IL-12* were similair between HP mice and controls [8]. However, we cannot exclude the possibility that these cytokines can increase after the normalization of IOP in RP mice.

## 5. Conclusions

In conclusion, to the best of our knowledge, this is the first study reporting that impaired vascular function and autoregulation due to increased IOP persist after IOP normalization. However, persistent vascular dysfunction does not promote further RGC loss. Oxidative stress and inflammation seem to be involved in the onset of vascular endothelial dysfunction and pericyte loss in the retina, but do not appear to contribute to its maintenance. Importantly, after IOP normalization, endothelial dysfunction does not appear to reduce the viability of RGCs and their axons.

## Data Availability

Data are contained within the article.
